# Use of red seaweed phytochemicals-zeolite nanocomposite as a feed additive to reduce ruminal methane emissions in vitro

**DOI:** 10.1007/s11250-025-04501-9

**Published:** 2025-06-13

**Authors:** Amira Othman, Taha Taha, Moamen Al-Shafii, Toka Deskoy, Mina Ratib, Mario Youssef, Eman Mohammed, Yosra Ahmed Soltan

**Affiliations:** Animal and Fish Production Department, Faculty of Agriculture, Alexandria University, Alexandria, Egypt

**Keywords:** Clays, Fermentation, Phytochemicals, Monensin, Zeolite-based nanocomposites

## Abstract

Natural alternative products to antimicrobials may offer a cost-effective and environmentally friendly substitute for conventional ionophore antibiotics as dietary feed additives to reduce methane (CH_4_) emissions from ruminants. This study is designed to prepare and assess the physicochemical properties and biological effects of red seaweed (*Asparagopsis taxiformis*) phytochemicals-zeolite nanocomposite (ZRN) in comparison to ionophore monensin and natural zeolite on ruminal fermentation. The wet impregnation technique was employed to combine the active components of red seaweed with zeolite to create ZRN. An in vitro gas production (GP) study was conducted to evaluate the biological impact of different levels of the developed ZRN on ruminal fermentation compared to monensin and natural zeolite. The experimental treatments included a control group (0 supplementations), monensin (40 mg/kg dry matter (DM) monensin), natural zeolite (20 g/kg DM natural zeolite), and ZRN were supplemented at 0.25, 0.5, and 0.75 g/kg DM ZRN to the control basal substrate. The experimental ZRN contained 27 highly active phytochemicals, such as 1,2-Benzene dicarboxylic acid, quercetin, and patchouli alcohol. Particle size distribution analysis revealed that the particle size at D90 decreased from 334 nm in natural zeolite to 46 nm in ZRN. The innovative ZRN exhibited a larger specific surface area, higher cation exchange capacity, and distinct morphology observed through electron microscopy compared to natural zeolite. All experimental feed additives reduced CH_4_ production compared to the control, with ZRN diets had the lowest (*P* = 0.02) CH_4_ values among all diets. A linear reduction effect of the ZRN prototype on ruminal GP (*P* = 0.007) and linear and quadratic reductions in CH_4_ production (*P* < 0.05) were observed, without adverse effects on organic matter degradability. ZRN supplementation increased (*P* < 0.05) ruminal pH and tended (*P* = 0.08) to decrease ammonia production compared to the control diet. Monensin showed a tendency towards reducing protozoal count (*P* = 0.08), while ZRN treatment resulted in linear and quadratic increases (*P* < 0.05). No differences were detected in total short-chain fatty acids among the experimental treatments. Significant increases (*P* = 0.018) were observed in the molar proportions of propionate due to monensin, whereas all treatments involving ZRN led to a significant increase (*P* = 0.001) in the molar proportions of acetate over propionate. These results indicate the successful preparation of the ZRN with enhanced physicochemical properties and biological effects for reducing CH_4_ production while promoting microbial fermentation. Thus, it could be considered as a novel dietary feed additive for dairy ruminant diets.

## Introduction

Most nations have established goals to reduce greenhouse gas (GHG) emissions by the second half of the twenty-first century (Belanche et al. 2023). Methane (CH_4_) is a particularly potent GHG that significantly contributes to climate change. It possesses a considerably higher global warming potential than carbon dioxide (CO_2_) over a shorter time frame (IPCC 2019). Furthermore, the loss of 2–12% of gross energy in the form of CH_4_ from ruminant animals represents inefficiency in feed energy utilization. Thus, it is crucial to consider this when aiming to improve feed efficiency and potentially redirect this energy towards milk or meat production (Belanche et al. 2023; IPCC 2019). To address this challenge, many antimicrobial feed additives, including ionophore antibiotics (as monensin), have been widely used as antimicrobial growth promoters to reduce CH_4_ emissions from dairy animals while enhancing their production performance and overall health status (Patra et al. 2017). It has high antimicrobial activity as it primarily disrupts bacterial cell wall membranes through ion exchange processes, particularly involving H +/Na + and H +/K + antiport activities (Patra et al. 2017). However, the use of these additives has raised concerns about the presence of residues in animal products (milk or meat), prompting a shift towards the exploration of natural products with high cation exchange capacity (CEC) that are safe for both animals and humans (Al Adawi et al. 2024).

Clays (e.g., zeolite) are widely recognized as safe for human and animal consumption (El-Nile et al. 2023). Natural zeolite clay is composed of aluminosilicates with potent antimicrobial agents and is known for its high CEC and strong sorption properties (Valpotić et al. 2017; Hamd et al. 2022). It has been extensively studied as a potential feed additive due to its ability to regulate ruminal pH, serve as an effective pH-buffering agent, and promote microbial ruminal fermentation without adverse effects on nutrient degradability (Soltan et al. 2022), however its natural form has lower efficiency in mitigating rumen CH_4_ emissions than ionophore antibiotics (Soltan et al. 2021a; El-Nile et al. 2021, 2023).

Recently, enhancing the antimicrobial activity of clays through modification to form nanocomposites has emerged as a promising strategy for developing feed additives aimed at reducing CH₄ emissions from ruminants. In the modified nanocomposite, one or more phases of the modifiers are dispersed within a matrix at the nanoscale, allowing molecular or atomic-level interactions that result in properties significantly different from those of the original clay before modification. Therefore, clay nanocomposites exhibited exceptional physicochemical properties, including higher CEC, increased intensity of functional groups (e.g., hydroxyl H–O–H and Si–O–Si bonds) and specific surface area, and enhanced adsorptive properties compared to the natural clay form (Al Adawi et al. 2024; Umejuru et al. 2023; Soltan and Patra 2024). For example, Soltan et al. (2021a) found that the nano compost of montmorillonite clay modified with surfactants as sodium dodecyl sulfate or cetyltrimethyl ammonium bromide, when supplemented at 0.5 g/kg DM, reduced CH_4_ formation by 38% and 57%, respectively, while natural montmorillonite and monensine reduced CH_4_ by only 6.8% and 28.3%, respectively compared to a control diet without compromising diet degradability in vitro.

Although these modified clay nanocomposites have been utilized to reduce CH_4_ emissions, they have typically been developed using synthetic chemical surfactants. But nowadays, the preference for natural phytochemicals over synthetic chemicals is driven by considerations of safety, biodegradability, reduced toxicity, consumer preference, pharmacological properties, environmental concerns, and regulatory requirements across various applications (Patra et al. 2017). Therefore, various advancements in phytochemical-clay nanocomposites can be developed to improve clays'physicochemical properties and thier antimicrobial activity (Umejuru et al. 2023). Hamd et al. (2022) developed a green seaweed-zeolite nanocomposite with efficient and eco-friendly adsorbent possesses antimicrobial activity due to its high ion exchange capacity, resulting in an impressive removal rate of 91.1% when applied in the removal of dyes from industrial wastewater. Al Adawi et al. (2024) reported that the Arabic gum-montmorillonite nanocomposite clay has demonstrated high antimicrobial activity due to enhancements in its physicochemical parameter [such as the presence of potent antimicrobial components (α-amyrin and lupeol) and functional groups like OH and Si–O] compared to the natural montmorillonite. These characteristics have led to significant improvements in the ruminal fermentation profile, CH_4_ inhibition, nutrient digestibility, milk yield, and health status of dairy cows.

In the current study, we hypothesized that combining well-known anti-methanogenic phytochemicals with zeolite in the form of nanocomposites could result in novel properties, providing safe alternative feed additives capable of reducing ruminal CH_4_ emissions while maintaining ruminal pH and nutrient degradability, thus we used red seaweeds *(Asparagopsis taxiformis)* extact as a modifier for zeolite, as it is known for their high potent anti-methanogenic properties but associated with a decrease in ruminal pH and consequently the fiber degradability (Machado et al. 2014, 2018; Paul et al. 2006; Roque et al. 2021; Choi et al. 2022). Therefore, the hypothesis of this study was that nano modifying zeolite with *A. taxiformis* phytochemicals would create a red seaweed-zeolite nanocomposite (ZRN) capable of serving as an antimicrobial alternative feed additive to monensin ionophore. This ZRN could potentially reduce ruminal CH₄ emissions while maintaining ruminal pH and nutrient degradability. Nevertheless, this is the first study to investigate ZRN and its impact on ruminal methanogenesis. Therefore, the objectives of this study were to develop ZRN as a natural altranative feed additive, assess its physicochemical properties, and evaluate its effects on ruminal fermentation, CH_4_ mitigation, and degradability in vitro.

## Materials and methods

This study was conducted at the Laboratory of Nanotechnology and Greenhouse Gasses, Faculty of Agriculture, Alexandria University, Alexandria, Egypt.

### Red seaweed and zeolite sources

The red seaweed (*A*. *taxiformis*) was collected during the rainy season at the gametophyte phase from the Mediterranean Sea coast between Alexandria and Rosetta, Egypt. The red seaweed was verified by the City of Scientific Research and Technological Applications in Borg El-Arab, Egypt. After harvesting, the seaweed underwent a thorough rinse with tap water. Subsequently, it was subjected to a drying process at 40 °C for a duration of 72 h. Following this, the seaweed was finely ground through a 0.25-mm screen and subjected to ethanol extraction. Concisely, 100 g of the finely ground red seaweed underwent extraction using a hydro-ethanolic solution (700 ml/l) with a ratio of 1000 ml at a temperature of 40 °C for a duration of 72 h. The resulting extract was then filtered through Whatman No. 1 filter paper (Camlab, Cambridge, UK). The filtrate obtained was subsequently evaporated at 45 °C until complete dryness was achieved, and it was stored at −15°C until the chemical analysis (Soltan et al. 2021b). Commercial natural raw zeolite (Ca, K_2_, Na_2_, Mg) 4 A _l8_ Si_40_ O_96_. 24 H_2_O) was provided from Yemen zeolite (Yemen Quarries & products, industry, investment Co. Ltd., Sanaa, Republic of Yemen).

### Preparation of red seaweed- zeolite nanocomposite

The ZRN was developed following the steps of the wet impregnation technique as described by Mosavi et al. (2021) but with some modifications. The required quantities of the natural zeolite and ethanolic extract of red seaweed (in a ratio of 1:1) were mixed in 20 ml of distilled water and magnetically stirred at 500 rpm for 60 min at 40° C using a stirrer with a hot plate (508- Hotplate- Magnetic, Globe Scientific, NY, USA). The mixture was ultrasonically treated for 60 min using a prop sonication (Model USY2500-1, 2500 W, Bioevopeak, China). This treatment was repeated two times. The resulting nanocomposite was filtered, washed with distilled water three times, and finally dried in an oven at 50 °C for 48 h to obtain the final experimental ZRN.

### Bioactive components of ZRN

The phytochemical components of the ZRN were identified using a Thermo Scientific TRACE-1300 series gas chromatography/mass spectrometry (GC/Mass; Thermo Fisher Scientific Inc., Waltham, MA, USA). The Mass spectra were scanned at the range of 40–700 amu, and the scan time was 5 scans/s. The chemical components of the experimental red seaweed were detected by the combination of retention index data with mass spectra data using the Mainlib library (Soltan et al. 2021b).

### The physicochemical properties of the experimental clays

The pH and electrical conductivity (EC) of both the natural zeolite and the modified ZRN were assessed separately. This evaluation was conducted in a suspension containing these feed additives and distilled water (with an initial pH of 6.81) at a ratio of 1:2.5. A multi-parameter pH meter (GLP 21 model; CRISON, Barcelona, Spain) was employed for these measurements. The cation exchange capacity (CEC) of the experimental feed additives was determined by using solutions containing 1 M sodium acetate and 0.1 M sodium chloride (Soltan et al. 2021a). To identify the elemental contents of the experimental feed additives, samples of natural zeolite and ZRN were analyzed using the energy-dispersive X-ray system (EDX) attached to a scanning electron microscope (SEM; Jeol JSM-6360 LA, 3–1–2 Musashino, Akishima, Tokyo, Japan). To examine the shapes of the red seaweed-zeolite nanocomposite and the unprocessed zeolite, SEM was employed. Before imaging, the samples were coated with a layer of gold to enhance the visualization of the sample specimen, as described in Soltan et al. (2021a).

The size distribution and specific surface area (SSA) of the natural zeolite and ZRN feed additives were determined through the utilization of a laser particle analyzer. The specific instrument employed for this analysis was the Better size 2600 particle analyzer (Dandong Bettersize Scientific Ltd. in Dandong, China). This instrument was equipped with an automated laser centering function to enhance accuracy.

### In vitro assay

#### The substrates and treatments

The basal diet was formulated to fulfill the nutritional needs of small ruminants following the guidelines provided by the National Research Council (NRC 2007). Subsequently, a sample of the diet was subjected to a comprehensive chemical analysis. The components analyzed included dry matter (DM, ID no. 930.15), organic matter (OM, ID no. 942.05), ether extract (EE, ID no. 945.38), and crude protein (CP, ID no. 954.01), and the analysis methods employed were in accordance with AOAC (2006) standards. The sequential determination of neutral detergent fiber (NDF), acid detergent fiber (ADF), and acid detergent lignin (ADL), excluding residual ash, was performed using filter bags (F57- ANKOM Technology Corporation, Macedon, USA) and a semi-automatic fiber analyzer unit (Smart Fiber analyzer, FA-12, Cairo, Egypt). This analysis procedure adhered to the methodology described by Van Soest et al. (1991). The composition of the basal diet, including its chemical constituents, is detailed in Table 1.

**Table 1:** Ingredients and chemical composition based on dry matter (DM) of the experimental basal substrate (50% concentrate: 50% forage) used in the in vitro experiment

	Experimental basal substrate
Item	(g/kg DM)
Ingredients	
* Trifolium alexandrinum* clover	455
Wheat straw	45
Ground maize	90
Wheat bran	95
Soybean meal	40
Barley	212
Brocken rice	45
Calcium carbonate	8
Sodium chloride	5
Vitamins and minerals mixture^1^	5
Chemical composition	
Organic matter	910
Crude protein	168
Neutral detergent fiber	480
Acid detergent fiber	137
Acid detergent lignin	128
Ether extract	15.0

The experimental treatments were planned as follows: a control group (0 supplementation), monensin [40 mg/kg DM monensin (Rumensin®, Elanco, Itapira, São Paulo, Brazil with 100 mg/kg purity)], natural zeolite (20 g/kg DM natural zeolite), and ZRN were supplemented at 0.25, 0.5, and 0.75 g/kg DM ZRN to the control basal substrate. The experimental dosage of monensin was determined according to the manufacturer's recommendations. This monensin dosage had previously been shown to effectively reduce CH_4_ production in vitro without adverse effects on ruminal nutrient degradability (Soltan et al. 2021a). Additionally, the experimental dose of natural zeolite was based on the study conducted by El-Nile et al. (2021).

#### Procedure for the gas production

The comparative assessment of the biological activity among the experimental monensin, ZRN, and natural zeolite was conducted employing the semi-automatic gas production (GP) technique as outlined in Bueno et al. (2005). To prepare the ruminal inoculum for the GP assay, samples of ruminal contents were collected individually from three buffalo calves with an average body weight of 400 ± 15.8 kg standard deviation. These animals underwent a fasting period before slaughter at the Faculty of Agriculture experimental station, at Alexandria University. These calves had been given unrestricted access to a locally sourced commercial diet (50% forage: 50% concentrate) formulated for beef production during a flushing period (approximately three weeks). The collected ruminal contents were promptly transferred to pre-warmed (39 °C) insulated flasks under anaerobic conditions, then homogenized using a blender, filtered through three layers of cheesecloth, and saturated with CO_2_.

The experimental substrate samples weighing 0.5 g each were placed into 120 ml serum flasks (*n = *12/treatment; Arab Pharmaceutical Glass Inc., Suez, Egypt) and incubated with 45 ml of diluted rumen fluid (comprising 15 ml of ruminal inoculum and 30 ml of Menke's buffered medium) (Soltan et al. 2024). After filling, all flasks were sealed with rubber stoppers, crimped using aluminum seals, and shaken before being placed in an incubator set at 39 °C. The identical procedure was employed for blank flasks (comprising a buffer solution and rumen inoculum), as well as for internal standard flasks (which included a buffer solution, rumen inoculum, and Egyptian berseem clover hay). The blank flasks were utilized to determine the net GP values, while the internal standard flasks were used to account for sensitivity variations introduced by the inocula. The gas headspace (75 ml) pressure was recorded at 3, 6, 9, 12, and (hours after incubation using a pressure transducer and data logger (model PSI-V2, SmartLab Inc., Cairo, Egypt).

#### Total gas and methane production

The gas pressure was converted to gas volume by using the following formula: GP (ml) = 6.5465 × p—0.9573 (*n = *600; *R2 = *0.99), where GP represents the gas volume in milliliters, and p denotes the measured pressure in psi. For the analysis of CH_4_, a one-milliliter sample of the headspace gas was collected at each pressure measurement time using a 3 ml syringe and then transferred to 5 ml vacutainer tubes. Methane concentrations were determined by a laser gas Pro detector (Gas-Pro- IR-W368334/01–001, Crowcon Detection Instruments Ltd, Abingdon, United Kingdom). Prior to CH_4_ determination, the detector was promptly calibrated according to the LRQA-validation ISO9001 quality manual, using a standard calibration gas mixture prepared following BS EN ISO 6145–1–2008.

#### Ruminal nutrient degradability, fermentation, and protozoal count

To inhibit the ruminal microbial activity, all incubation flasks were immediately transferred to an ice-cold environment (0 °C) after the 24-h incubation period. Flasks designated for measuring nutrient degradability underwent treatment with a neutral detergent solution for 3 h at 90 °C, following the methodology outlined by Blümmel et al. (1997). The residual materials that had not undergone degradation within the incubation flasks were collected in pre-weighed clean crucibles, subjected to washing with hot distilled water and acetone, and subsequently dried at 70 °C for a period of 48 h. The dried residues were then ashed at a temperature of 600 °C for 2 h. The calculation of true degraded organic matter (TDOM) and true degraded neutral detergent fiber (TDNDF) involved determining the differences between the quantities of material incubated and the quantities that remained non-degraded, a methodology previously detailed (Bueno et al. 2005). The partitioning factor (PF) was calculated as the ratio of TDOM (mg) and volume of GP (ml) (Blümmel et al. 1997).

The contents of the flasks that were customized for measuring the fermentation parameters and protozoal count were centrifuged at 10, 000 g for 15 min, and the supernatant was used to measure the individual short-chain fatty acids (SCFA) and ammonia concentrations. The measurement of ruminal pH values was conducted with a pH meter (CRISON GLP2, Barcelona, Spain). To determine ruminal NH_3_-N concentrations, a spectrophotometer (E-2100 model; PEAK Instruments; Houston; USA) was employed, and this was carried out using a commercially available laboratory kit (Biodiagnostic Inc., Giza, Egypt) at a wavelength of 635 nm via calorimetric analysis. The quantification of total protozoa was achieved through microscopy, following the procedure detailed by Dehority et al. (1983), using a bright-line hemacytometer counting chamber.

For the determination of SCFA, a 1.6 ml aliquot of the supernatant was combined with 0.4 ml of (250 g/l) metaphosphoric acid in a 4:1 ratio. This mixture was then centrifuged at 15,000 rpm for 20 min at 4 ºC using a K1015 Micro Prime centrifuge (Centurion Scientific Ltd, Stoughton, Chichester, UK). Concentrations of SCFA were assessed based on the method outlined by De Baere et al. (2013) with some adaptations. High-performance liquid chromatography (HPLC) equipment was employed, specifically the Agilent 1260 Infinity II system with a Quaternary Pump (Agilent Technologies, Inc., Santa Clara, California, USA). The detection was carried out using an Agilent 1260 Infinity Multiple Wavelength Detector equipped with a 1024-element photodiode array. The HPLC system included an autosampler (model: 1260 Infinity II Vialsampler) and a separation column (Eclipse XDB-C18, 4.6 × 150, 3.5µm Rapid Resol; Agilent Technologies, Inc., Santa Clara, California, USA). Calibration of the integrator was performed using a mixture of known SCFA concentrations as an external standard (Sigma Chemie GmbH, Steinheim, Germany).

### Statistical analysis

All data obtained from the in vitro assay were analyzed using the MIXED procedure of SAS (SAS Institute Inc., Cary, USA, version 9.0), incorporating a one-way ANOVA design. The experimental unit was defined as the incubation bottle. Experimental parameters were analyzed as part of a completely randomized design with repeated measures, utilizing the following statistical model: Yij = μ + Di + Aj + eij, in which Yij represents the observation, μ stands for the overall mean, Di accounts for the fixed effect of treatment, Aj represents the random effect associated with the incubation bottle, and eij denotes the random residual error. Orthogonal contrast statements were formulated to evaluate the linear and quadratic responses of each dependent in vitro parameter as they related to increasing levels (0, 0.25, 0.5, and 0.75 g/kg DM diet) of the ZRN. Statistical significance was determined at P ≤ 0.05, and trends were noted at P ≤ 0.10."

## Results

### GC–MS analysis of ZRN

The experimental ZRN contained 27 phytochemicals with different concentrations and functional groups. Highly active components like 1,2-Benzene dicarboxylic acid, quercetin [4H-1-Benzopyran-4-one,2-(3,4-dihydroxy phenyl)], 4 h-1-benzopyran-4-one, 2-(3,4-dihydroxyphenyl)−6,8- di-á-d glucopyranosyl-5,7- dihydroxy, triamcinolone acetonide and patchouli alcohol were found in abundance in ZRN (Table 2).

**Table 2:** Chemical constituents identified by gas chromatography/mass spectrometry analysis in red seaweed- zeolite nanocomposite (ZRN)

Peaks	Compounds	RT (min)a	Peak area (%)	Molecular formula	Molecular weight
1	Methiopropamine 3'-thiophene isomer	13.72	1.80	C_8_H_13_NS	155
2	1,4-Benzenedicarboxylic acid,	14.95	5.83	C_18_H_16_O_6_	328
3	Propiolic acid, 3-(1-hydroxy-2-isopropyl-5-methylcy clohexyl)	17.21	1.74	C_13_H_20_O_3_	224
4	Spathulenol	18.71	1.81	C_15_H_24_O	220
5	Phen-1,4-diol, 2,3-dimethyl-5 trifluoromethyl-	19.11	0.69	C_9_H_9_F_3_O_2_	206
6	Cedrane, 8-propoxy	19.41	2.64	C_18_H_32_O	264
7	Aromadendrene oxide-(2)	19.84	1.17	C_15_H_24_O	220
8	2,7-Diphenyl-1,6-dioxopyridazino[4,5:2',3']pyrrolo[4',5'-d]pyridazine	20.05	1.34	C_20_H_13_N_5_O_2_	355
9	Propanoic acid, 2-methyl-, (dodecahydro-6a-hydroxy-9a-methyl- 3-methylene-2,9-dioxoazuleno[4,5-b]furan-6-yl)methyl ester	20.53	2.20	C_19_H_26_O_6_	350
10	Patchouli alcohol	20.78	4.60	C_15_H_26_O	222
11	4H-1-Benzopyran-4-one, 2-(3,4-dihydroxyphenyl)−6,8- di-á-d-glucopyranosyl-5,7- dihydroxy-	23.36	1.42	C_27_H_30_O_16_	610
12	Hexadecane, 1,1-bis(dodecyloxy)	24.43	3.78	C_40_H_82_O_2_	594
13	4H-1-benzopyran-4-one, 2-(3,4-dihydroxyphenyl)−6,8-di-α-D-glucopyranosyl-5,7-dihydroxy	24.52	1.23	C_27_H_30_O_16_	610
14	N-(2-{4,5-dimethoxy-2-[2-pheNylethenyl]phenyl}−3-phenYlpropyl)-n,n-dimethylami ne hydrochloride	25.70	2.44	C_27_H_32_ClNO_2_	437
15	Cyclopropanedodecanoic acid, 2-octyl-, methyl ester	26.32	2.30	C_24_H_46_O_2_	366
16	4 h-1-benzopyran-4-one, 2-(3,4-dihydroxyphenyl)−6,8- di-á-d glucopyranosyl-5,7- dihydroxy-	26.42	0.70	C_27_H_30_O_16_	610
17	2-acetyl-3-(2-benzenesulpho Namido)ethyl-7-methoxyin dole	27.71	2.71	C_19_H_20_N_2_O_4_S	372
18	Docosanoic acid, 8,9,13-trihydroxy-, methyl ester	29.41	1.18	C_23_H_46_O_5_	104
19	4H-1-benzopyran-4-one, 2-(3,4-dihydroxyphenyl)−6,8-di-α-D-glucopyranosyl-5,7-dihydroxy	29.56	10.49	C_27_H_30_O_16_	610
20	Androstan-17-one, 3-(formyloxy)−11-hydroxy (3à,5à,11á)	30.04	2.43	C_20_H_30_O_4_	334
21	4H-1-Benzopyran-4-one,2-(3,4-dihydroxy phenyl)	30.58	9.23	C_15_H_10_O_7_	302
22	2-Hydroxy-3-[(9e)−9-octadec enoyloxy]propyl (9e)−9 octadecenoate	33.56	8.28	C_39_H_72_O_5_	620
23	Tetraneurin—a -diol	33.61	9.31	C_15_H_20_O_5_	280
24	Triamcinolone Acetonide	35.09	5.39	C_24_H_31_FO_6_	434
25	Ursodeoxycholic acid	35.62	2.66	C_24_H_40_O_4_	392
26	1,2-Benzene dicarboxylic acid	36.64	10.88	C_24_H_38_O_4_	390
27	Flavone 4'-oh,5-oh,7-di-o-glucoside	38.09	3.16	C_27_H_30_O_15_	594

^a^RT retention time

### The physicochemical properties of the experimental feed additives

The measured physicochemical properties of the resultant ZRN differed from the natural zeolite. Negligible variations in pH were detected between the modified and natural zeolite clays, while the modified ZRN presented higher electrical conductivity than the natural zeolite. Zeolite-red seaweed nanocomposite presented a higher CEC and specific surface area than the natural zeolite (Table 3).

**Table 3:** Physicochemical properties of the unmodified natural zeolite and the developed prototype of red seaweed- zeolite nanocomposite (ZRN)

	Zeolite clays	
Items	Natural Zeolite	ZRN
pH	9.00	8.7
Electrical conductivity (ppm)	266	744
Cation exchange capacity (meq/100 g)	100	126.1
Specific surface area (m^2^/kg)	8188	50,622

**Fig. 1:**
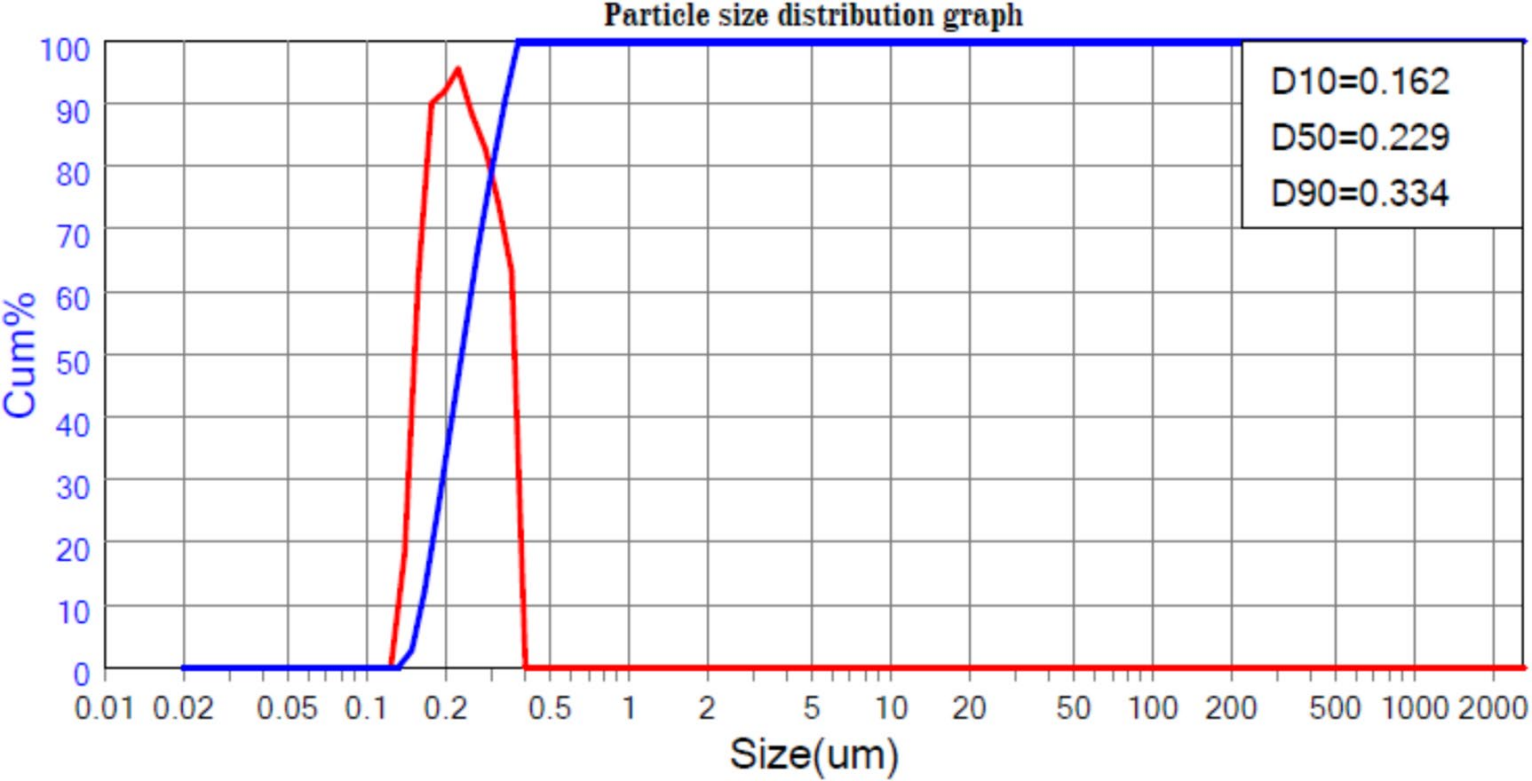
Particle size analysis of the experimental

**Fig. 2:**
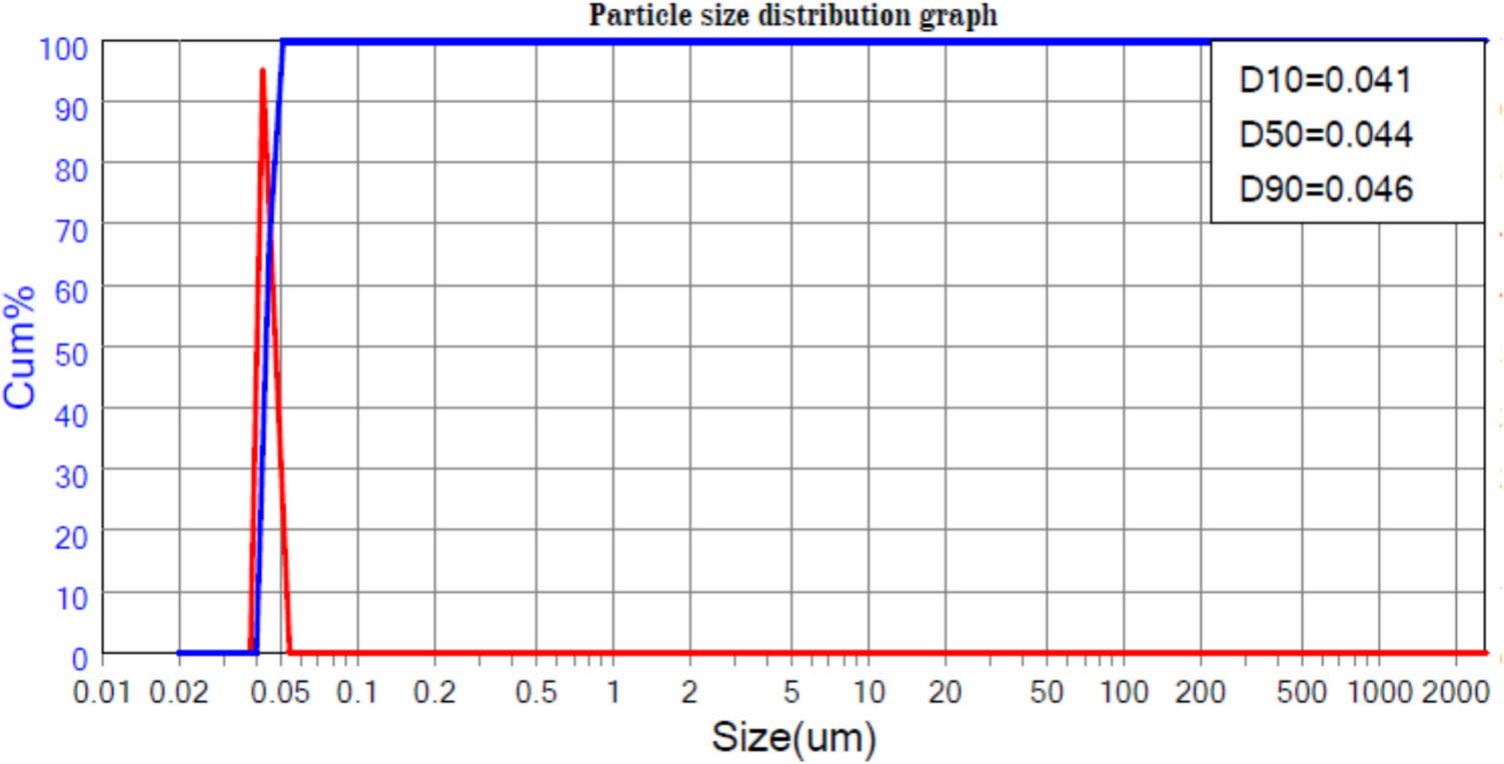
Particle size analysis of the experimental prototype of red seaweed- zeolite nanocompost (ZRN) natural zeolite

Figures 1 and 2 show the particle size distribution analysis for both natural zeolite and the experimental ZRN. The results indicate a successful transformation from a broad distribution in the case of natural zeolite clay (ranging from 162 to 334 nm) to a narrower distribution for ZRN in the nanoscale range (ranging from 41 to 46 nm). The particle size at D90 decreased from 334 nm in natural zeolite to 46 nm in ZRN.

**Fig. 3:**
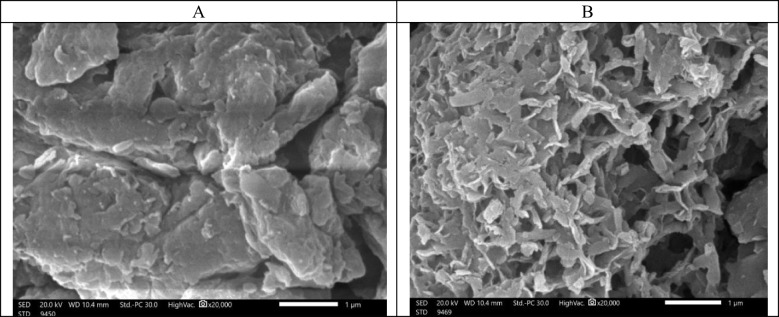
Scanning electron microscope (SEM) analysis of the natural zeolite (A) and the experimental prototype of red seaweed−zeolite nanocomposite (B)

**Fig. 4:**
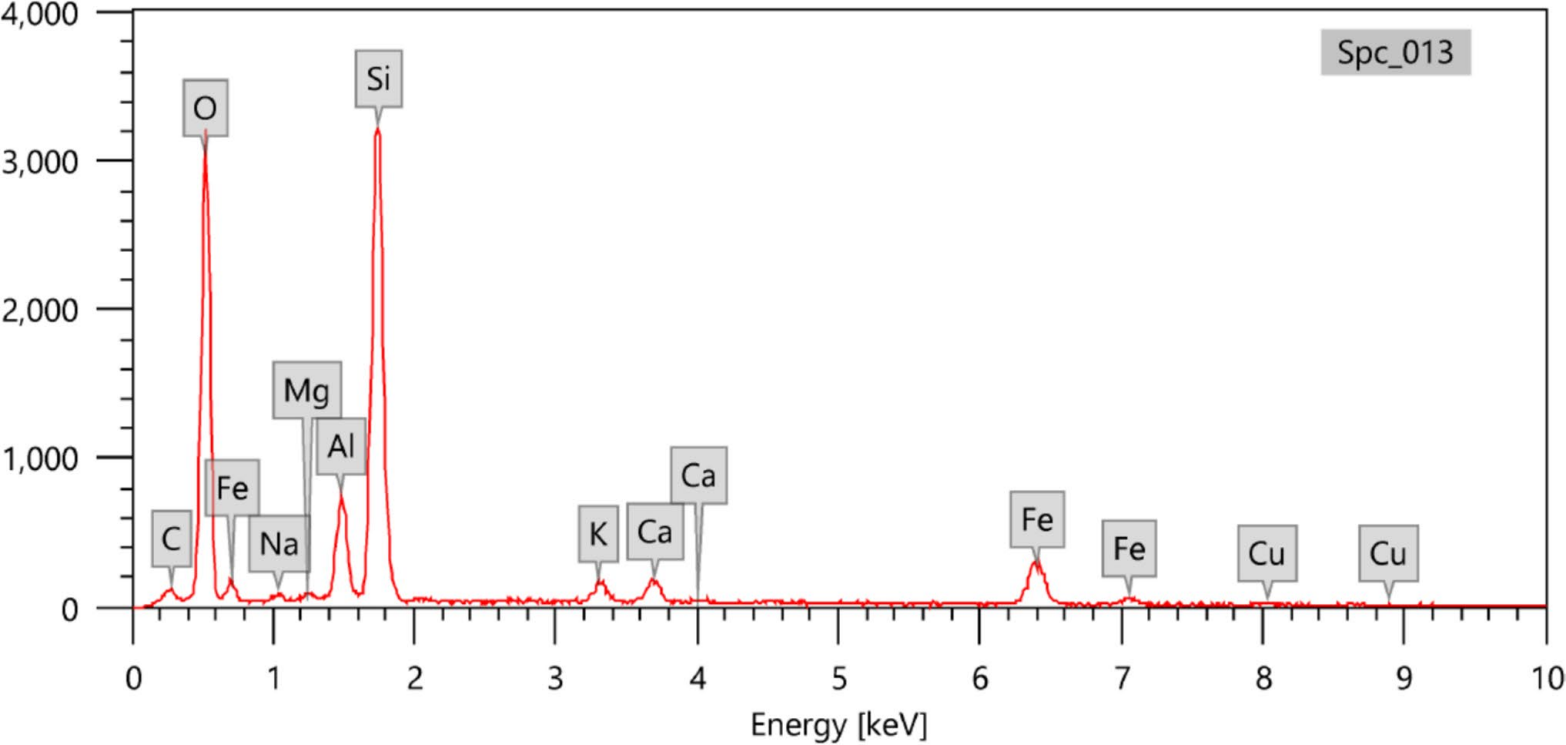
Energy dispersive X-ray (EDX) for the devloped zeolite-red seaweed nanoparticles (ZRN)

**Fig. 5:**
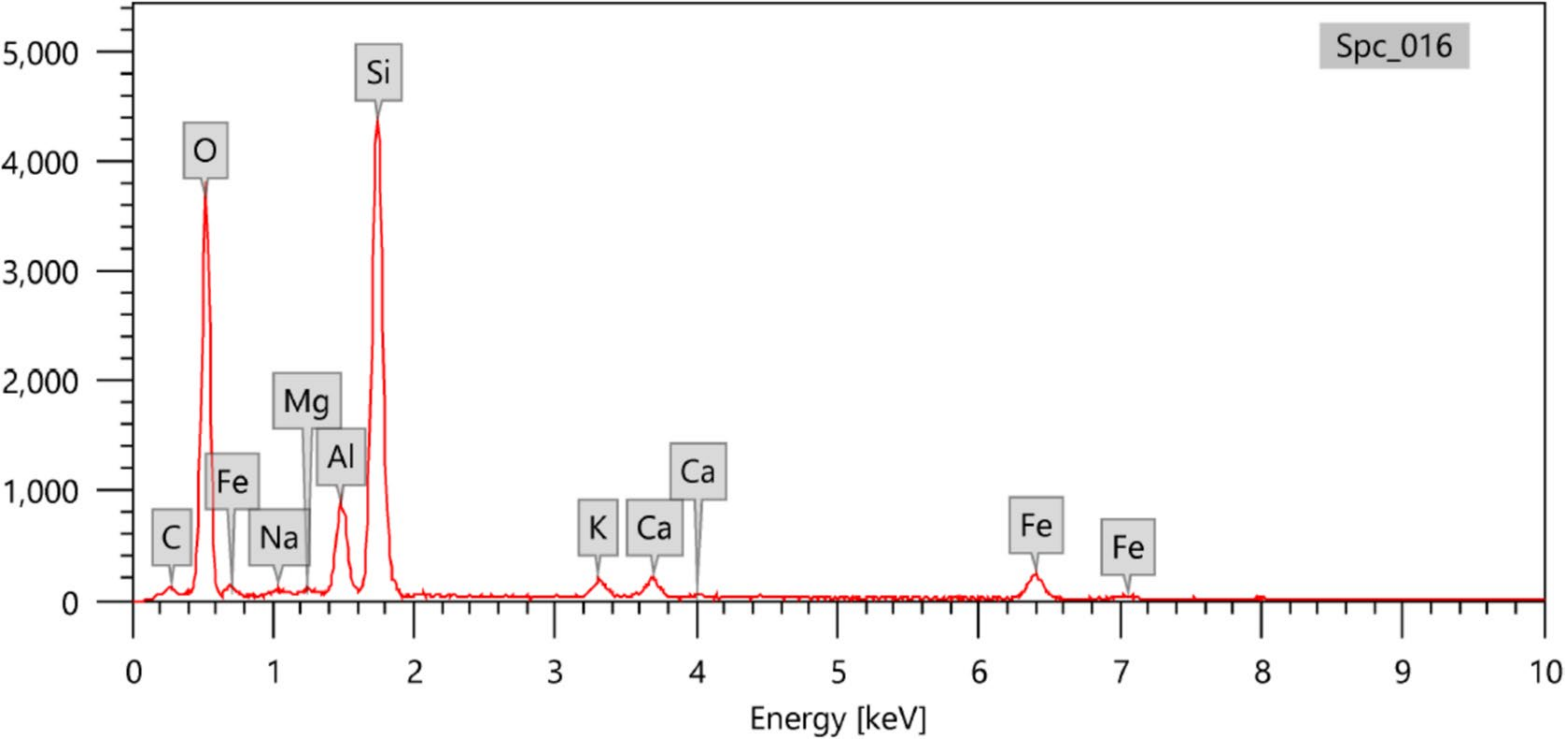
Energy dispersive X-ray (EDX) for the natural zeolite

Figure 3 shows the SEM analysis of the natural zeolite (A) and the experimental prototype of ZRN. This analysis reveals that the edges of the ZRN flakes became cracked and exhibited a rough appearance compared to the natural zeolite. Figures 4 and 5 show the EDX for the ZRN and natural zeolite, respectively. High concentrations of Si were detected in the natural zeolite compared to the natural zeolite, while Cu was only detected in the prepared ZRN structure.

Ruminal gas and CH_4_ production, nutrient degradability, and PF.

All doses of ZRN reduced (*P* = 0.002) GP compared to the control. All the experimental feed additives decreased CH_4_ production compared to the control, and ZRN diets presented the lowest (*P* = 0.02) CH_4_ (ml/g TDOM) values compared to all diets. The contrast analysis showed a significant linear decreasing effect of the ZRN on ruminal GP (*P* = 0.007) and linear and quadratic reduction effects on CH_4_ production (*P* < 0.05). No adverse effects (*P* > 0.05) were observed on the TDOM or fiber degradability among the experimental treatments, while a quadratic reducing effect (*P* = 0.03) was observed by the ZRN doses on TDNDF values. A significant linear increasing effect (*P* < 0.001) was observed on the PF values by increasing the experimental dose of our innovative ZRN feed additive. Both monensin and ZRN supplemented at 0.75 g/kg DM showed a similar tendency for reducing ammonia concentration after 24 h of incubation (*P* = 0.08) compared to other diets, with no contrasting effects observed for ammonia values with different ZRN doses (Table 4).

**Table 4:** Supplementation effects of monensin, natural Zeolite and red seaweed- zeolite nanocomposite (ZRN) on ruminal gas production, methane emission (CH_4_), true degraded organic matter (TDOM), true degraded neutral detergent fiber (DNDF) and partitioning factor (PF)

Items	Treatments (T)							*P* value		
	Control	Monensin	Zeolite							
			Natural	ZRN					Contrast	
				ZRN_1_	ZRN_2_	ZRN_3_	SEM	T	Linear	Quadratic
Gas production (ml/g IOM)	203^a^	190^ab^	187^ab^	183^b^	173^b^	174^b^	4.837	0.002	0.007	0.982
CH_4_										
(ml/g IOM)	11.56^a^	9.54^ab^	9.59^ab^	8.173^b^	8.51^b^	8.65^b^	0.521	0.006	< 0.001	0.005
(ml/g TDOM)	14.6^a^	13.83^b^	12.5^b^	10.92^c^	11.07^c^	11.05^c^	0.789	0.020	0.008	0.02
Nutrient degradability (g/kg DM)										
TDOM	778	777	767	756	769	782	14.11	0.799	0.866	0.046
DNDF	604	569	549	527	553	577	29.30	0.565	0.586	0.031
PF (mg TDOM/ml gas)	3.81	3.76	3.556	3.743	4.17	4.146	0.204	0.287	< 0.001	0.635

SEM standard error of the mean, ZRN_1_ = ZRN supplemented at 250 mg/kg dry matter, ZRN_2_ = ZRN supplemented at 500 mg/kg dry matter, ZRN_3_ = ZRN supplemented at 750 mg/kg dry matter, Contrast: = effects of control (0 supplementation mg/kg dry matter) compared with ZRN_1_, ZRN_2,_ and high ZRN_3_

a,b,c,d Means within a row without a common superscript letter differ significantly at P < 0.05

### Ruminal fermentation and protozoal count

The diet supplemented with the highest dose of ZRN prototype (700 mg/kg DM) resulted in a significantly higher (*P* = 0.0005) ruminal pH compared to the diets of control, monensin, and the natural zeolite. A quadratic increasing effect (*P* = 0.04) on ruminal pH was observed with ZRN supplementation. Furthermore, ZRN supplementation led to significant increases (*P* < 0.05) in ruminal pH and a tendency (*P* = 0.08) to decrease ammonia production compared to the control diet. Monensin showed a tendency towards reducing protozoal count (*P* = 0.08), while ZRN treatment resulted in both linear and quadratic significant effects (*P* < 0.05) (Table 5).

**Table 5:** Supplementation effects of monensin, natural Zeolite and red seaweed- zeolite nanocomposite (ZRN) on ruminal pH, ammonia concentration and protozoal count total short-chain fatty acids (SCFAs) concentration (mM), and molar proportions of individual SCFAs (% of total SCFA)

Items	Treatments (T)							*P* value		
	Control	Monensin	Zeolite							
			Natural	ZRN					Contrast	
				ZRN_1_	ZRN_2_	ZRN_3_	SEM	T	Linear	Quadratic
Ruminal pH	5.86^b^	5.056^c^	5.81^b^	6.78^ab^	6.74^ab^	6.136^a^	0.22	0.0005	0.117	0.046
Ammonia (mg/100 ml)	19.5	16.43	16.83	17.5	17.2	16.2	0.74	0.083	0.010	0.542
Protozoal count × 10^5^	6.90	5.60	7.30	6.20	8.60	6.99	1.28	0.085	<.0001	0.0001
SCFAs										
Total (mM)	106	147	137	160	102	156	16.55	0.116	0.0782	0.978
Acetate (mol/100 mol)	51.9^bc^	50.5^c^	55.4^abc^	61.1^a^	58.8^ab^	60.8^a^	1.622	0.001	<.0001	0.0003
Propionate (mol/100 mol)	26.1^ab^	31.9^a^	25.6^ab^	20.47^b^	25.2^ab^	20.9^b^	2.006	0.018	0.0253	0.4849
Butyrate (mol/100 mol)	20.5^a^	14.99^b^	16.7^b^	15.7^b^	13.3^b^	15.16^b^	0.737	0.0004	<.0001	<.0001
Valerate (mol/100 mol)	0.986	2.02	1.652	2.098	1.506	2.247	0.324	0.137	0.0267	0.866
Isovalerate (mol/100 mol)	0.378	0.459	0.308	0.577	0.61	0.77	0.1847	0.543	0.0011	0.343

SEM standard error of the mean, ZRN_1_ = ZRN supplemented at 250 mg/kg dry matter, ZRN_2_ = ZRN supplemented at 500 mg/kg dry matter, ZRN_3_ = ZRN supplemented at 750 mg/kg dry matter, Contrast: = effects of control (0 supplementation mg/kg dry matter) compared with ZRN_1_, ZRN_2,_ and high ZRN_3_

a,b,c,d Means within a row without a common superscript letter differ significantly at *P* < 0.05

The results of the SCFA profile indicated that ZRN had a different effect on ruminal fermentation compared to natural zeolite and monensin. However, no significant differences (*P* > 0.05) were observed in total SCFA among the experimental treatments. Notably, significant increases (*P* = 0.018) were observed in the molar proportions of propionate due to monensin, whereas all treatments involving ZRN led to a significant increase (*P* = 0.001) in the molar proportions of acetate over propionate. The control had significantly higher (*P* = 0.0004) butyrate molar proportions compared to all the feed supplementations. Furthermore, the contrast analysis revealed that ZRN had both linear and quadratic decreasing effects (*P* < 0.05) on butyrate concentrations, while neither the treatments nor the contrast analysis indicated differences (*P* > 0.05) in the valerate and isovalerate molar proportions (Table 5).

## Discussion

Many of the phytochemicals identified in ZRN were found to possess a range of health benefits and practical applications, including antioxidant, antimicrobial, and anti-inflammatory properties. For instance, 1,2-Benzene dicarboxylic acid was screened for its antimicrobial activity against eleven human pathogenic bacteria (Waheed et al. 2019). Quercetin, also known as 4H-1-benzopyran-4-one, 2-(3,4-dihydroxyphenyl), is a flavonoid recognized for its antioxidant and antimicrobial attributes (Oskoueian et al. 2013). When quercetin combined with naringin at a concentration of 45 g/kg of the substrate (on DM), it demonstrated the potential to inhibit CH_4_ production without causing any adverse effects on rumen microbial fermentation (Oskoueian et al. 2013). The literature also pointed out the antimicrobial and antioxidant properties of components that contain glucopyranosyl in its structure as 4 h-1-benzopyran-4-one, 2-(3,4-dihydroxyphenyl)−6,8- di-á-d glucopyranosyl-5,7- dihydroxy that occurred in abundance in our prototype (Hassan et al. 2008). Detection of these active components of ZRN may confirm the suitability of the wet impregnation technique to achieve high loading levels of the red seaweed extract on the zeolite, providing control over the surface properties and functionality of the resulting ZRN material. The binary and tertiary combinations of various plant secondary metabolites can significantly enhance the bioactivities of these mixtures by leveraging their antioxidant and antimicrobial effects, ultimately influencing the microbial fermentation profile (Soltan et al. 2021b).

The combination of zeolite and red seaweed extract using the wet impregnation technique resulted in a narrower size distribution, a reduction in particle size, and an increase in the specific surface area of the prepared ZRN compared to natural zeolite. The increase in the specific surface area of ZRN can be attributed to the reduction in particle size induced by the presence of nanoparticles. This enhancement in surface area allows for more significant exposure to chemical reactions (El-Nile et al. 2021). As nanoparticles possess a large surface-to-volume ratio, enhancing their bioactivity and rendering them effective bactericidal agents (El-Nile et al. 2021). This improvement is further supported by the higher CEC observed in ZRN compared to natural zeolite. A higher CEC indicates a greater number of metal hydrolysates and ions that can be incorporated into the clay interlayer space, ultimately contributing to the improved activity of the prepared ZRN (Xue et al. 2007).

The SEM and EDX analyses can validate the successful modification of zeolite clay by red seaweed extract. The SEM images reveal that the edges of the ZRN flakes exhibit cracks and a rough appearance compared to natural zeolite. This change primarily results from the separation between the clay layers (Soltan et al. 2022), induced by the presence of red seaweed extract settled between the silica tetrahedron layers of the zeolite. Furthermore, the EDX analysis indicates the presence of Cu and alterations in Si content in ZRN relative to natural zeolite, providing supporting evidence for this assertion (Ullah et al. 2022). These findings also imply that ZRN may demonstrate enhanced antibacterial activity compared to natural zeolite, attributable to the incorporation of Cu within its structure. The effectiveness and mechanism of Cu's antimicrobial activity against microorganisms have been extensively documented in various literature sources (Salah et al. 2021). This multifaceted process primarily hinges on the generation of reactive oxygen species (ROS), which inflict irreversible damage upon membranes (Salah et al. 2021).

This is the first study conducted to assess the impact of different levels of our ZRN feed additive on ruminal fermentation, in comparison to monensin and natural zeolite. Consequently, challenges may arise when seeking to make direct comparisons between our findings and those of similar studies. The current results confirm that ZRN can indeed exert beneficial effects on modulating ruminal fermentation patterns, resulting in reduced CH_4_ production. However, it should be noted that this effect was associated with a quadratic reduction in total GP and NDF degradability. These findings suggest that careful consideration must be given to selecting the optimal supplementation doses of ZRN to maximize the nutritional benefits obtained from its addition. The literature on various red seaweed extracts has consistently shown a significant reduction in CH_4_ production. This reduction is often accompanied by decreased DM degradability, both total and individual SCFA, as well as a decrease in GP after 12 h of the in vitro incubation (Choi et al. 2022). However, no adverse effects were observed in the current study of the SCFA or TDOM by the experimental ZRN. The literature has reported that red seaweed can reduce CH_4_ formation through specific anti-methanogenic phytochemicals such as bromoform and dibromochloromethane (Paul et al. 2006; Machado et al. 2014). However, these components were not detected in our ZRN prototype. It was reported that red seaweed *(A. taxiformis*) exhibits a more effective reduction in CH_4_ compared to equivalent amounts of pure bromoform, likely because multiple anti-methanogenic CH_4_ analogs within the seaweed work synergistically (Machado et al. 2018). Therefore, it seems that the reduction in CH_4_ caused by ZRN may not be solely attributed to these phytochemicals. Other phytochemicals detected in ZRN may also contribute to the CH_4_ reduction. Zeolite may also contribute to this reduction, suggesting a combined antimicrobial effect that exceeds that of natural zeolite. This could be attributed to the high CEC, increased specific surface area, heightened activity resulting from the size-quantization effect of ZRN, and the presence of Cu in its structure (El-Nile et al. 2021).

One of the notable findings in the current study is that the experimental ZRN showed superior efficacy in reducing CH_4_ compared to monensin. Indeed, ZRN could be acknowledged as a novel methanogen inhibitor. However, the precise factors contributing to this reduction require further exploration, given the current absence of information regarding the effects of ZEN on ruminal bacteria or methanogens. Monensin is known as a modifier of ruminal fermentation through its antimicrobial effects which are attributed to its CEC properties, resulting in a reduction in bacterial intracellular K + and an increase in intracellular Na +, as demonstrated by Russell and Strobel (1989) and Schären et al. (2017). It is noteworthy that this action has a more pronounced detrimental effect on gram-positive bacteria compared to gram-negative bacteria, as indicated by Calsamiglia et al. (2007). This selective inhibition leads to a shift in the rumen's bacterial composition, which can confer nutritional advantages in ruminants. Gram-positive bacteria are key contributors to the production of hydrogen which is main the substrate for methanogenesis (Calsamiglia et al. 2007). By suppressing these bacteria, monensin reduces the availability of hydrogen for methanogenic archaea, redirecting hydrogen utilization away from formate and methanogenesis toward the production of alternative hydrogen-sink products, such as succinate and propionate, which are more energetically favorable, thereby lowering CH_4_ production (Russell and Strobel 1989). In this study, the typical mode of action to reduce CH_4_ emissions by monensin was observed. It involved promoting a shift in the SCFA pattern towards greater proportions of propionate and concurrently reducing the acetate proportion and ruminal pH. However, despite these benefits, the use of ionophores raises significant concerns regarding antimicrobial resistance and concerns about the presence of residues in animal products. These issues have led to increased interest in finding natural alternatives that can achieve similar benefits without the associated risks (Al Adawi et al. 2024). Thus in our study, we investigated the potential of ZRN as a sustainable and safe alternative to traditional ionophores like monensin. Unlike monensin, ZRN shifted the pattern of SCFA production towards acetate rather than propionate. The reasons for this phenomenon are not clear; nevertheless, it could be speculated that the unique combination of zeolite and seaweed active components present in ZRN might influence ruminal microbes through more potent mechanisms of action, such as antimicrobial and antioxidant properties, potentially leading to enhances in acetate production, ruminal pH, and PF. The physicochemical properties of ZRN, as determined in our study, reveal high surface area and CEC as indicators for antimicrobial activity (Xue et al. 2007; El-Nile et al. 2021). Additionally, the antioxidant properties of ZRN phytochemicals (such as the presence of glucopyranosyl) would help to mitigate oxidative stress within the rumen (Hassan et al. 2008). These combined effects can selectively inhibit certain harmful or less efficient microbial populations, allowing more efficient fiber-degrading microbes, such as cellulolytic bacteria, to thrive. This shift in microbial population dynamics could enhance acetate production, as these efficient fiber-degrading microbes are primarily responsible for the breakdown of cellulose into acetate (Soltan et al. 2024, 2021b; Al Adawi et al. 2024). Furthermore, the stabilization of ruminal pH and improvement in partitioning factor (PF) observed in our study can be attributed to these antimicrobial and antioxidant effects, which together create a more favorable ruminal environment for acetate production.

The literature reports that robust radical scavenging activities in the individual components of red seaweed (Oskoueian et al. 2013). This characteristic serves to mitigate oxidative stress, thereby fostering more favorable conditions for microbial growth, which partly explains the increase in PF values observed by the ZRN treatments. Improvements in PF values (as an indicator of microbial protein synthesis) could potentially play a role in the observed reduction in CH_4_ by ZRN (Calsamiglia et al. 2007). The removal of H + from the rumen environment is known to elevate ruminal pH and stimulate microbial activity. Therefore, when CH_4_ levels decrease, H + ions may be utilized in the production of SCFA to ensure optimal ATP production for microbial mass growth at the expense of CH_4_ formation (Blümmel et al. 1997). An elevated ruminal pH can improve protein solubility and facilitate the production of branched-chain volatile fatty acids (Soltan et al. 2017). This partially explains the linear increase observed in isovalerate levels in the current study due to ZRN treatment. Increases in ruminal pH may also explain the enhancement in acetate production by ZRN compared to monensin. It is known that most ruminal acetate producers and protozoal populations are sensitive to acidic conditions, whereas acid-tolerant species are propionate producers (Soltan et al. 2021b). Therefore, the reduction in pH resulting from monensin administration may lead to a shift in SCFA production, favoring a higher propionate formation when compared to ZRN treatment. These results also explain the higher protozoa count and acetate caused by ZRN compared to monensin. It seems that the utilization of ZRN resulted in the suppression of CH_4_ formation by promoting acetate production. Acetate formation competes with CH_4_ production for available H + within the rumen (Soltan et al. 2017). When acetogens effectively convert hydrogen and carbon dioxide into acetate, there is a reduced amount of hydrogen available for methanogenesis, ultimately leading to decreased CH_4_ emissions (Schären et al. 2017). There is a wealth of literature confirming that acetogenins can play a role in reducing CH_4_ production from ruminants. Following the acetyl-CoA pathway (4H_2_ + 2 CO_2_ → CH_3_COOH + 2H_2_O), reductive acetogenesis presents a hydrogenotrophic process that can act as an alternative hydrogen sink to methanogenesis in the rumen (Gagen et al. 2015).

It has been reported that CH_4_ reduction can be achieved through either the inhibition of methanogen numbers or their activity or indirectly by reducing protozoal populations (Patra and Saxena 2010; Soltan et al. 2021a). Protozoa are known to provide methanogens with substantial substrates, such as hydrogen and formate, facilitating methanogenesis (Russell and Strobel 1989; Calsamiglia et al. 2007). However, in the current study, the reduction in CH_4_ caused by ZRN treatment did not coincide with a decrease in protozoa abundance. Therefore, it can be suggested that ZRN might directly inhibit methanogenesis. It is worth noting that we used ruminal inocula obtained from buffalo calves. Thus, it is crucial to acknowledge potential species-specific variations in microbial diversity, enzyme activity, and fermentation characteristics that may influence the interpretation and generalizability of our findings if other ruminal sources such as goats, sheep, or cows are considered (Calabrò et al. 2005).

## Conclusion

The modified zeolite in the form of ZRN prototype exhibited higher CEC values, an increased specific surface area, a narrower nano-size distribution, and contained biological phytochemicals (including 1,2-Benzene dicarboxylic acid and quercetin) compared to natural zeolite. Supplementation of ZRN at a level of 700 mg/kg emerges as a promising feed additive for effectively mitigating CH_4_ emissions while preserving superior effects over monensin or natural zeolite on rumen fermentation, microbial protein synthesis.

## Data Availability

All data of this manuscript are included in this article version.
